# H-NOX and NosP Regulate Flagellar Protein and Virulence Factor Production in *Vibrio Cholerae*

**DOI:** 10.1016/j.mcpro.2026.101654

**Published:** 2026-09-03

**Authors:** Thathmi Wathsara Herath Pathiranage, Md. Shariful Islam, Cedar Stellon, Michelle D. Cherne, Daniel Valencia, Diane Bimczok, Erik T. Yukl

**Affiliations:** 1 Department of Chemistry and Biochemistry, New Mexico State University, Las Cruces, New Mexico, USA; 2 Department of Microbiology and Cell Biology, Montana State University, Bozeman, Montana, USA; 3 Center for Biofilm Engineering, Montana State University, Bozeman, Montana, USA

**Keywords:** Vibrio, cholerae, phosphoproteome, bacterial signaling, motile/sessile transition, nitric oxide

## Abstract

The ability of *Vibrio cholerae* to transition between motile and sessile forms in the environment and in the host is critical to its survival and virulence. The molecular cues, sensor proteins, and signaling pathways mediating these transitions are highly complex and often overlapping. Nevertheless, a detailed understanding of them is critical for understanding the persistence and pathogenesis of this deadly pathogen. Nitric oxide functions as an important signaling molecule in many bacteria, affecting biofilm formation, motility, and virulence, often through interaction with heme protein sensors. The genome of *V*. *cholerae* encodes two such sensors called H-NOX and NosP. Here we constructed a *Δhnox/nosP* mutant and employed a multiomics methodology that combines tandem mass tag–based quantitative proteomics, phosphoproteomics, and targeted metabolomics to investigate the function of these sensors. A set of 258 proteins was differentially expressed in the mutant that included many proteins involved in flagellar biosynthesis and motility as well as critical virulence factors, iron acquisition systems, and metabolic enzymes. Many of the identified genes are also part of the ferric uptake regulator regulon and iron-dependent transcriptional repression of several ferric uptake regulator targets was disrupted. Phosphoproteomics analysis also revealed proteins involved in motility and virulence as differentially phosphorylated in the mutant strain. In most cases, these phosphoproteins have not been previously observed and provide a wealth of new targets for investigating mechanisms of *V. cholerae* signaling. Taken together, this work illustrates a role for H-NOX and NosP in promoting factors important for infection while suppressing those important for environmental survival, suggesting a function in priming the organism for infection and/or maintaining the infectious phenotype.

## Highlights

• A combined -omics approach highlights regulation in *Vibrio cholerae*.
• H-NOX and NosP are involved in sessile-motile transitions.
• New phosphorylated proteins are revealed in *V. cholerae*.

## Graphical Abstract

**Figure:**
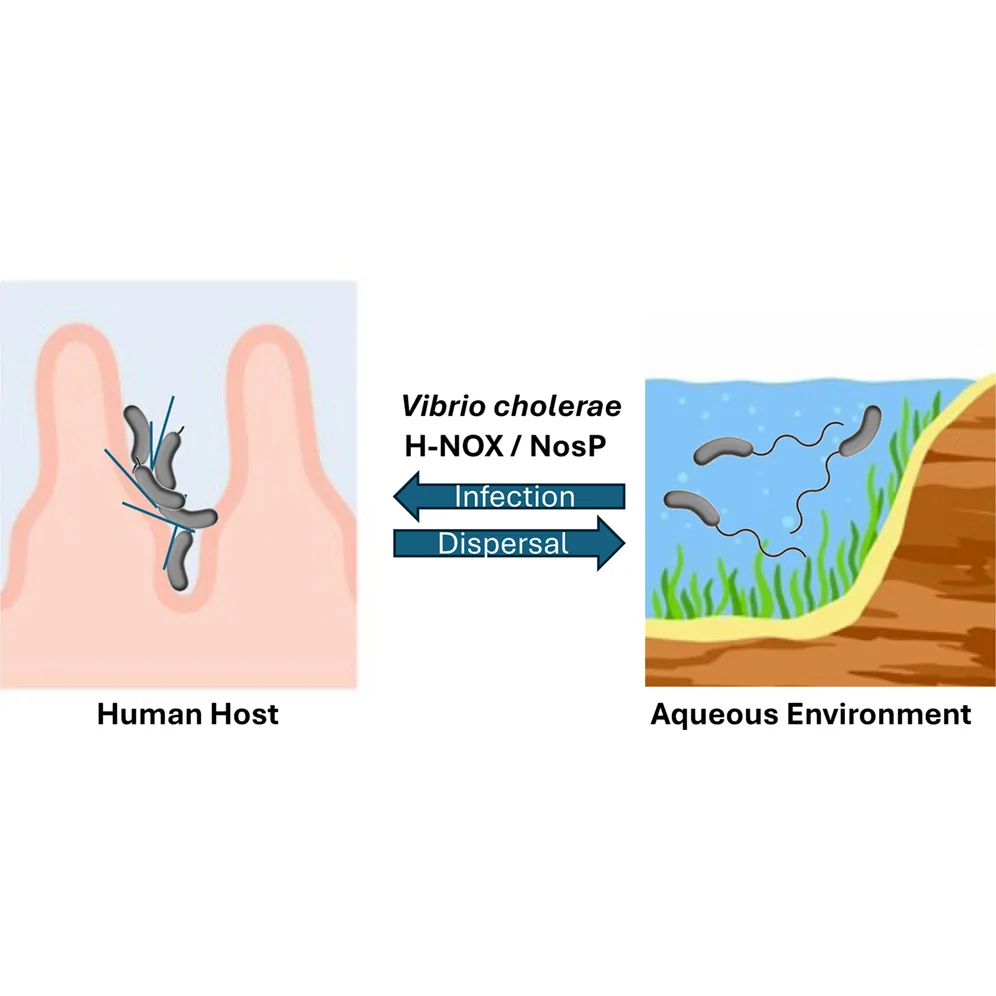

## In Brief

*Vibrio cholerae* is a deadly pathogen that must transition between motile and sessile lifestyles to complete its complex cycle of infection and environmental persistence. This work uses integrated omics to demonstrate that two sensor proteins, H-NOX and NosP, drive changes in protein expression promoting factors important for infection while suppressing those important for environmental survival. Such signaling mechanisms are potential targets for therapeutic interventions for the disease cholera.

*V. cholerae* is an environmental pathogen found in coastal and estuarine waters where it may exist in a planktonic state or in biofilms predominantly associated with the chitinous exoskeletons of zooplankton and small crustaceans (1). Biofilms not only protect this species from environmental stresses (2) and predation (3), but also present a hyperinfectious phenotype (4) upon ingestion by the human host. Once ingested, *V. cholerae* must colonize the surface of the small intestine, forming microcolonies that produce virulence factors such as the toxin-coregulated pilus (TCP) that facilitates colonization (5) and the cholera toxin (CtxAB) that induces constitutive cyclic AMP production in the host and causes the secretory diarrhea that represents clinical cholera (6). Finally, large numbers of bacteria detach from host cells to be shed in stool and facilitate transmission to other hosts. This complex cycle involves several transitions between motile and sessile lifestyles mediated by numerous environmental cues and molecular and physiological changes (for a comprehensive review, see (7)).

One such environmental cue regulating biofilm dynamics in multiple bacterial species is nitric oxide (NO) (8). NO is a cytotoxic agent used in animals as a defense against infection (9) as well as an important signaling molecule in both eukaryotes and prokaryotes (10, 11). Two hemoproteins, H-NOX and NosP, have been identified as important bacterial sensors for NO (see recent reviews (12, 13, 14)). Both hemoproteins are widely conserved (15, 16) and participate in metabolism of the secondary messenger cyclic diguanosine monophosphate (c-di-GMP) (17) and/or quorum sensing (18) pathways to regulate processes such as motility, biofilm formation, and virulence.

Most bacteria encode either H-NOX or NosP but not both. An exception to this is *V*. *cholerae*, where NO has been shown to lead to biofilm dispersal (19) through upregulation of flagellar biosynthesis genes in aflagellated biofilm-associated cells (20). The genome of *V. cholerae* serotype O1 encodes H-NOX, NosP, and CdpA, a multidomain protein containing a NosP domain. H-NOX exposed to NO or oxidants inhibits a histidine kinase HnoK (21), which phosphorylates several targets including a c-di-GMP phosphodiesterase (PDE) HnoB (22) (Fig. 1). NosP binds NO and inhibits another histidine kinase VpsS, which participates in a larger quorum sensing circuit (23) that ultimately regulates many diguanylate cyclase and PDE genes involved in c-di-GMP metabolism (24) as well as virulence factors such as TCP and CtxAB (25) (Fig. 1). CdpA has a C-terminal PDE domain whose activity is inhibited by heme binding to the NosP domain (26) but stimulated when NO binds to the heme (27). The resulting decrease in c-di-GMP reduces the activity of the pilus extension motor MshE, resulting in retraction of mannose-sensitive hemagglutinin pili and detachment from abiotic surfaces.

**Fig. 1:**
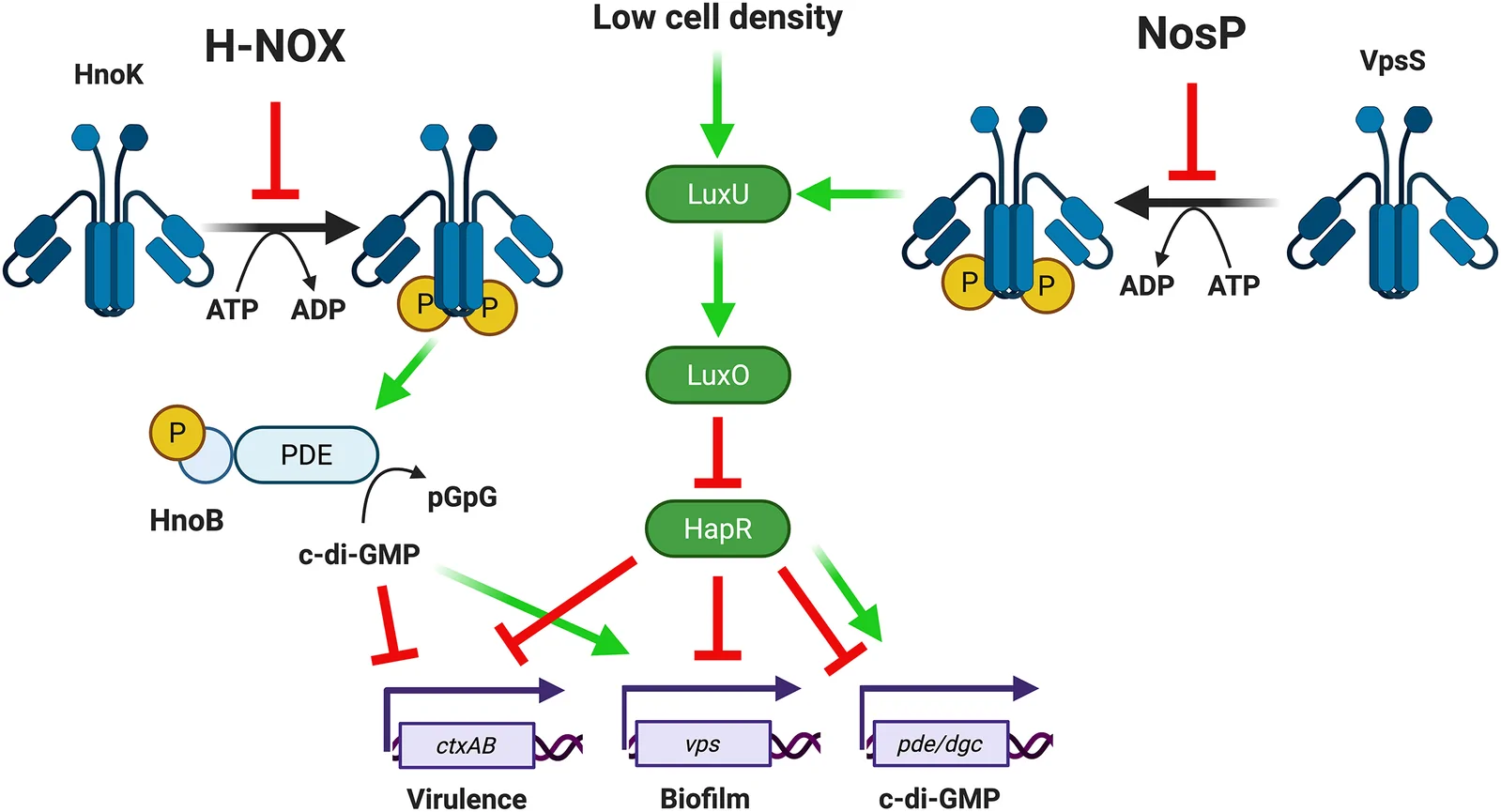
**H-NOX and NosP signaling in *Vibrio cholerae*.***Green arrows* indicate activating phosphorylation or gene induction events while *red hammers* indicate inhibitory processes. Modified from (28) and created in BioRender. Yukl, E. (2026) https://BioRender.com/v14p3lu. H-NOX, heme-nitric oxide/oxygen binding; NosP, nitric oxide-sensing protein.

All three *V. cholerae* heme-based sensor proteins likely participate in NO sensing and c-di-GMP metabolism, which is important for biofilm formation (29) and virulence (30). Their signaling pathways are complex and likely overlapping. Furthermore, to our knowledge, only *cdpA* has been deleted from the *V. cholerae* genome (27), facilitating a more detailed assignment of its function and molecular mechanism in this organism than is available for either H-NOX or NosP. Here we undertook the generation of Δ*hnox* and Δ*nosP* deletion strains as well as a Δ*hnox*/*nosP* mutant in *V. cholerae* serotype O1 classical Ogawa 395 (O395) strain. This strain produces the major virulence factors and, along with other classical strains, was responsible for early cholera pandemics (31). We took an unbiased approach to determining signaling pathways by using total proteomics to evaluate changes in protein abundance between strains grown under standard culture conditions in rich media. The results revealed unexpected connections between H-NOX/NosP signaling and the production of flagella and virulence factors, iron homeostasis, and central carbon metabolism. We evaluated the impact of these changes on growth and survival, iron-dependent gene expression, motility, and the relative abundance of central carbon metabolites. Phosphoproteomics was also undertaken, providing an extensive list of phosphoproteins in *V. cholerae* and demonstrating differential phosphorylation for several proteins involved in motility, chemotaxis, and virulence in the mutant strain. Taken together, this work suggests important and partially redundant functions for H-NOX and NosP in regulating virulence factors and the sessile-motile transition with likely consequences for infection and persistence within the environment.

## Experimental Procedures

### Experimental Design and Statistical Rationale

For microplate cell growth and quantitative reverse transcription polymerase chain reaction experiments three biological replicates (n = 3) and three technical replicates were run for a total of nine measurements. Proteome analyses were conducted on WT and mutant *V. cholerae* strains cultured in three (n = 3) biological replicates. Metabolomics experiments utilized four (n = 4) replicates and phosphoproteomics experiments utilized five (n = 5) to achieve sufficient statistical power. Proteins and metabolites with significantly differential abundance between strains were identified by applying a two-sided *t* test with a cutoff of log_2_ fold change greater than 0.58 and a *p* value less than 0.05. The sample preparation and data analysis sections provide a comprehensive discussion of the various sample preparation optimization experiments, growth conditions, MS parameters, and analysis parameters. The data were analyzed using python, Origin, and R for various statistical tests depending on the type of comparison being made. These tests included the two-tailed, unpaired Student's *t* test for pair-wise comparisons, the one-way ANOVA with Dunnett's multiple comparisons test for samples grouped by one factor, and the two-way ANOVA with Tukey's multiple comparisons test for samples grouped by two factors. The appropriate test was selected based on the specific analysis being performed. A significance level of less than 0.05 was considered to be statistically significant.

### Bacterial Strains, Plasmids, and Media

The bacterial strains and plasmids used in this study are listed in Supplemental Table S1. Primers are listed in Supplemental Table S2. Strains were maintained at −80 °C in Luria-Bertani (LB) plus 20% v/v glycerol. Antibiotics were used in following concentrations: ampicillin, 100 μg/ml; streptomycin, 100 μg/ml; gentamycin, 50 μg/ml; kanamycin, 50 μg/ml. *V. cholerae* strains were grown in LB media or M9 minimal media (MM) containing final concentrations of 171 μM EDTA, 300 μM CaCl_2_, 1.0 mM MgSO_4_, 0.4% w/v glucose, 0.08 μM MnSO_4_, 0.42 μM CoCl_2_, 0.76 μM CuCl_2_, 31 μM FeCl_3_ unless otherwise specified, and 6.2 μM ZnCl_2_. For growth on succinate, 10 or 50 mM succinate was substituted for glucose. *V. cholerae* and *E. coli* were grown with consistent shaking at 30 °C and 37 °C respectively. P_BAD_ promoter-containing plasmids were induced by addition of 0.1 to 0.2% arabinose.

### Construction of *V. cholerae* Mutant Strains

Mutants were generated using homologous recombination through sucrose counterselection, as described previously (32, 33). KO plasmids were constructed using suicide vector pGP704:sacB, carrying upstream and downstream flanking regions of *hnox* (VC0395_0659), *nosP* (VC0395_A1054), or *nadC* (VCO395_0108). The *E. coli* SM10 λpir strain was used to transfer the DNA by conjugation to *V. cholerae*. Merodiploids resulting from a single crossover were selected on ampicillin and streptomycin (to which *V. cholerae* O395 is natively resistant), followed by sucrose counterselection. Attempts to generate Δ*hnox/nosP* double KOs by introducing a deletion of the second target gene into a single KO background were unsuccessful, reverting to the WT genotype at the second target locus in more than 100 cases. To overcome this, *hnox* on a pBAD18-kan arabinose-inducible plasmid (34) was introduced into the Δ*hnox/nosP* merodiploid *via* electroporation. Transformants were selected on ampicillin, streptomycin, and kanamycin containing 0.1% w/v arabinose followed by counterselection on sucrose and kanamycin containing 0.1% w/v arabinose. The resulting Δ*hnox*/*nosP* strain carrying the *hnox* gene in pBAD18-kan was cured of the plasmid by multiple passages in LB media lacking kanamycin followed by replica plating on LB-agar containing streptomycin and streptomycin plus kanamycin. The resulting kanamycin sensitive, Δ*hnox*/*nosP* strain was used for further studies.

All strains were analyzed by whole genome Illumina sequencing (SeqCoast Genomics, Supplemental Dataset 1), which revealed a third deletion of 14 base pairs near the 5′ end of the Divalent Anion Sodium Symporter gene *nadC* (VC0395_0108) in both kan-resistant and kan-sensitive Δ*hnox*/*nosP* strains. To determine whether the *nadC* deletion is a suppressor of lethality in Δ*hnox*/*nosP*, the *nadC* deletion was complemented in the Δ*hnox*/*nosP* strain by providing it in *trans* on a pBAD-kan plasmid as for *hnox*. RT-PCR confirmed inducible expression by arabinose, yet no change in growth was observed compared to the WT or parent Δ*hnox*/*nosP/nadC* (Δ*xpc*) strain (Supplemental Fig. S1). The genome of *V*. *cholerae* O395 encodes two other Divalent Anion Sodium Symporter proteins (VC0395_A0932 and VC0395_A2740) with 30% and 29% sequence identity to NadC, respectively, suggesting a high degree of functional redundancy. Consistent with this, growth in media with succinate as the sole carbon source was unimpaired in the Δ*xpc* mutant (Supplemental Fig. S2). Finally, a Δ*nadC* strain was generated and transcription of several genes differentially regulated in the Δ*xpc* strain were compared to WT (Supplemental Fig. S3). No significant differences were observed, confirming that these changes are due to deletion of *hnox* and *nosP*. Taken together, we conclude that the *nadC* deletion was a random mutation acquired during *hnox*/*nosP* deletion and unlikely to participate in H-NOX/NosP signaling. Nevertheless, this mutant will be referred to as Δ*xpc* to reflect its actual genotype.

### Bacterial Growth and Survival

Cultures were grown aerobically in LB medium with shaking at 30 °C until OD_600_ reached approximately 2.0. These were used to inoculate 200 μl of culture media in 96-well microplates to an OD_600_ of 0.05. The plates were sealed with gas permeable sealing membranes (Sigma-Aldrich). Cells were grown in LB or MM media at 30 °C for 24 h with hourly shaking at 200 rpm using BioTek EPOCH 2 plate reader. After 24 h, cells were diluted and plated on LB-agar to determine colony forming units. For stress tolerance assays, LB medium was supplemented with 0.1 to 1.5 mM DETA-NONOate or paraquat. DETA-NONOate is an NO donor with a very slow half-life. Based on previous estimates (35, 36), these concentrations are expected to yield steady-state NO concentrations in the low to mid-nanomolar range. For iron limitation experiments, cultures were grown in MM growth medium with added ferric iron concentrations of 30 μM, 3 μM, and 0 μM.

### Metal Content Analysis

*V. cholerae* WT and Δ*xpc* were grown overnight in LB medium with shaking at 30 °C. A total of 1 ml of culture was washed once with LB or MM containing various concentration of iron, resuspended in the same media, and grown overnight with shaking at 30 °C. These were used to inoculate larger cultures to an OD_600_ of 0.05 and were grown until OD_600_ *∼* 0.5. Approximately 10 to 15 ml of cells were pelleted at 4000*g* for 15 min and washed three times with 1 x PBS and dried at 105 °C to a constant weight. The dry cell pellet was digested in 250 μl of concentrated HNO_3_ for 5 h at 70 °C. Digested cells were diluted to 2.5 ml with milliQ water, and metal content was analyzed using PerkinElmer 2100 DV inductively coupled plasma-optical emission spectrometer. Multielement standard (Ricca Chemical) was used to calibrate the instrument, samples were run in triplicate, and cellular metal was expressed as nanograms of metal per milligram of dry cell weight.

### Proteomic and Phosphoproteomic Sample Preparation

All strains were grown to OD_600_ *∼* 0.5 with shaking at 30 °C in LB media. The cells were centrifuged at 14,000*g* for 10 min, and cell pellets were lysed in radioimmunoprecipitation buffer (Pierce) containing protease inhibitor in a ratio of 1:100. The cell lysates were homogenized by passing through a gauge 21 needle three times, followed by centrifugation at 14,000*g* for 10 min and the supernatants were collected. The protein concentration was measured using Bradford assay and approximately 90 μg of protein was used for further processing. The protein sample was brought to 100 μl with HPLC-grade H_2_O. Reduction was performed using tris(2-carboxyethyl) phosphine-dihydrochloride at 37 °C for 30 min. Alkylation was carried out by adding iodoacetamide to final concentration of 30 mM followed by 60 min incubation at RT in dark. Chloroform-methanol extraction was conducted by adding methanol and chloroform followed by centrifugation at 140,000*g* for 5 min. The resulting top aqueous layer was removed, and methanol was added, then the mixture was centrifuged. The pellet obtained was air dried and subjected to trypsin digestion by adding 100 mM triethylammonium bicarbonate and 2 μg of trypsin (sequencing grade modified, Promega). The digestion was carried out at 37 °C for 18 h, and desalting was performed using Sep-Pak C18 1 cc Vac Cartridge (Waters).

Phosphoproteomic analysis was performed only for the WT and Δ*xpc* strains. Phosphatase inhibitor (PhosSTOP, Roche) was added to the radioimmunoprecipitation buffer along with the protease inhibitors, and approximately 300 μg of protein was used. The reduction, alkylation, chloroform/methanol extraction, and desalting steps were carried out as previously.

Digested peptides were labeled using a tandem mass tag (TMT) 16-plex isobaric label reagent set (Thermo Fisher Scientific) and combined into one multiplex sample group. For phosphoproteomic analysis, peptides were labeled using a TMT 10-plex isobaric label reagent set (Thermo Fisher Scientific), combined into a single multiplex group, then enriched using High-Select TiO_2_ and Fe-NTA phosphopeptide enrichment kits (Thermo Fisher Scientific) following the manufacturer’s instructions. The labeled peptides were separated into 46 fractions on a 100 × 1.0 mm Acquity BEH C18 column (Waters) with the use of an UltiMate 3000 UHPLC system (Thermo Fisher Scientific). The process was carried out with a 50 min gradient, ranging from 99:1 to 60:40 buffer A:B ratio (Buffer A = 10 mM ammonium hydroxide, 0.5% acetonitrile, Buffer B = 10 mM ammonium hydroxide, and 99.9% acetonitrile).

The resulting superfraction was then further separated by reverse phase XSelect CSH C18 2.5 μm resin (Waters) on an in-line 150 × 0.075 mm column using an UltiMate 3000 RSLCnano system (Thermo Fisher Scientific). Peptides were eluted using a 75 min gradient from 98:2 to 60:40 buffer A:B ratio (Buffer A = 0.1% formic acid, 0.5% acetonitrile, Buffer B = 0.1% formic acid, 99.9% acetonitrile). Eluted peptides were ionized by electrospray (2.2 kV) followed by mass spectrometric analysis on an Orbitrap Eclipse Tribrid mass spectrometer (Thermo Fisher Scientific) using multinotch MS3 parameters. MS data were acquired using the FTMS analyzer in top-speed profile mode at a resolution of 120,000 over a range of 375 to 1500 m/z. Following CID activation with a normalized collision energy of 31.0, MS/MS data were acquired using the ion trap analyzer in centroid mode and normal mass range. Using synchronous precursor selection, up to 10 MS/MS precursors were selected for HCD activation with a normalized collision energy of 55.0, followed by acquisition of MS3 reporter ion data using the FTMS analyzer in profile mode at a resolution of 50,000 over a range of 100 to 500 m/z.

### RT-PCR Quantification

Cultures were grown to OD_600_ = 0.5 to 0.6 in LB or MM. A total of 300 ul of 5% (v/v) phenol in ethanol was added to 1 ml of bacterial culture, incubated in ice for 30 min, centrifuged, and the cell pellet stored at −80 °C. The RNA was extracted using Pure Link RNA Mini Kit (Invitrogen) and on-column DNAse treatment was used to remove DNA contaminants. Superscript III first-strand synthesis kit (Invitrogen) was used to synthesize complementary DNA from 1 μg of RNA. cDNA was diluted to 10 ng/μl and used for real-time PCR. The primers (Supplemental Table S2) were designed using the PrimerQuest tool (Integrated DNA Technologies) with annealing temperature ∼55 °C to amplify 100 to 120 bp of target genes and used at a final primer concentration 0.4 μM. SYBER green PCR master mix (Applied Biosystems) was used for amplification and reactions were monitored by CFX-Connect real-time system (Bio-Rad). Relative expression levels were calculated using the ΔΔC_t_ method and normalized to GAPDH expression (37).

### Motility Assays

*V. cholerae* were labeled with GFP by insertion of the *gfp* gene into a Tn7 site in the *V. cholerae* genome as previously described (38) with some modifications. Briefly, the pTn7-gfp plasmid (Supplemental Table S1) harboring the *gfp* gene was transferred to *V. cholerae* WT and deletion strains by triparental mating among *V. cholerae*, *E. coli* SM10-λ*pir* carrying pUX-BF13, and *E. coli* SM10-λ*pir* carrying pTn7-gfp. Exconjugants were selected on thiosulfate-citrate-bile salts-sucrose agar plates with gentamicin (50 μg/ml). The GFP-tagged strains were selected *via* colony PCR, and GFP expression was confirmed using flow cytometry.

Spontaneous motility of WT and Δ*xpc* GFP-*V. cholerae* was quantified using videomicroscopy. *V. cholerae* were streaked on agar plates and incubated overnight at 37 °C. For motility experiments, colonies were transferred into LB broth and diluted to achieve an OD_600_ = 0.02. The cell suspension (30 μl per well) was then transferred into 96-well glass bottom plates, and cultures were immediately imaged for 10 s on an inverted Keyence (Itasca, IL) BZ-X810 fluorescent microscope at 30 frames per second using a GFP-filter cube and a 60X objective. Keyence ∗.avi files were converted into a series of ∗.png images using the BZ-X800_OME-TIFF converter package. Motility features were obtained using Imaris software (Oxford Instruments), and data were analyzed using GraphPad Prism version 10.6.1.

### Metabolomics Sample Preparation

The method was adapted from a previously modified protocol in our laboratory (39). Briefly, overnight cultures were inoculated to OD_600_ = 0.05 in 10 ml of LB medium and grown to OD_600_ = 0.5 to 0.6. Cells were centrifuged at 100,000*g* for 10 min and washed three times with ice-cold 1 X PBS. The PBS washed cells were resuspended in 1 ml of ice-cold methanol containing 1 μM of internal standards, L-methionine sulfone and 2-morpholinoethanesulfonic acid. The suspension was homogenized by passing through a gauge 21 needle three times. The cell lysate was centrifuged at 18,400*g* at 4 °C for 10 min and supernatant was collected. The supernatant was extracted to deionized water:chloroform in a 1:2 ratio and centrifuged at 15,000 rpm at 4 °C for 15 min. The aqueous layer was collected and filtered using a 3 kDa molecular weight cutoff filter (Millipore). The filtered samples were lyophilized and stored in −80 °C until analysis. The dried samples were resuspended in water and analyzed *via* Shimadzu triple quadrupole mass spectrometer (LCMS-8050).

### Triple Quadrupole Mass Spectrometry Analysis

The analysis was performed using Primary Metabolites LC/MS/MS method package developed by Shimadzu with minor modifications (39, 40). Briefly, extracts were analyzed using a Shimadzu LCMS-8050 instrument which couples LC with a triple-quadrupole MS instrument. LC column, Mastro 2 (Shimadzu, C18, 3 μm, 150 × 2 mm) was used and the mobile phases were water (A: 15 mM acetic acid, 5 mM tributylamine-water) and methanol (B). The LC gradient program was as follows: 0% B (0.5 min) - 25% B (8.0 min) - 98% B (12.0–15.0 min) - 0% B (15.1–20.0 min). Flow rate was 0.30 ml/min and the column temperature was kept at 40 °C throughout the run. Nebulizing and drying gases were set to 2.0 and 10 L/min, respectively. The desolvation line and heat block temperatures were kept at 250 °C and 400 °C, respectively. The LCMS-8050 was operated in negative (−) mode electrospray ionization with multiple-reaction-monitoring (MRM) for each of the compounds as described in the Primary Metabolites package. The metabolites were normalized to cell mass.

### Omics Data Analysis

*V. cholerae* cells were cultured to the exponential growth phase and harvested to investigate the impact of Δ*xpc* on the global proteome, metabolome, and phosphoproteome. Extracted proteins were labeled using TMT and analyzed by LC–MS/MS. Protein identification and quantification of MS^3^ reporter ions were performed using MaxQuant (version 2.2.0.0) (41) with the *V. cholerae* UniProtKB database (UP000000249), comprising 3774 protein entries. The parent ion mass tolerance was set to 3 ppm, fragment ion tolerance to 0.5 Da, and reporter ion tolerance to 0.003 Da. A maximum of two missed cleavages was allowed during database searching. Carbamidomethylation of cysteine residues was specified as a fixed modification, whereas methionine oxidation and protein N-terminal acetylation were set as variable modifications. For phosphoproteomic analyses, phosphorylation of serine, threonine, tyrosine, histidine, and aspartate residues were included as variable modifications.

Peptide validation and protein identification were carried out using Scaffold Q + S software with false discovery rates of 1% at both the peptide-spectrum-match and protein levels. Only proteins identified by at least two unique peptides were retained. Protein probabilities were assigned using the Protein Prophet algorithm (42). Normalization of TMT MS^3^ reporter ion intensities was performed using ProteinNorm (43) with the variance stabilizing normalization method (44). Statistical analyses were conducted using the Linear Models for Microarray Data (limma) framework with Empirical Bayes (eBayes) moderation for standard-error estimation (45). Proteomic and metabolomic datasets were each generated from four independent biological replicates per condition, while phosphoproteomic datasets used five replicates. Comparisons between WT and Δ*xpc* strains were assessed using two-tailed Student’s t-tests. Metabolites and proteins were considered significantly altered at *p* < 0.05 and |log_2_ fold change| ≥ 0.58 (1.5-fold change). All analyses and visualizations were performed in Python 3.10 using the NumPy, pandas, Matplotlib, and Seaborn libraries.

## Results

### Global Proteome Profiling of Mutant and WT Strains

To explore the global proteomic alterations linked to the deletion of *hnox* and *nosP*, we conducted TMT-based quantitative proteomics on WT, Δ*hnox*, Δ*nosP*, and double mutant (referred to throughout as Δ*xpc*, see Experimental procedures) strains, identifying 2812 proteins. Comparison of WT with Δ*hnox* revealed only 36 differentially expressed proteins (2 upregulated and 34 downregulated) and these at very modest fold changes (Supplemental Dataset S2, Supplemental Fig. S4, *A* and *B*). To capture the overall structure of the dataset and visualize proteome-wide variation between conditions, we applied Uniform Manifold Approximation and Projection (UMAP) for nonlinear dimensionality reduction. This analysis did not demonstrate a robust separation between WT and Δ*hnox* samples, illustrating their very similar proteome distribution. Comparison of WT and Δ*nosP* revealed more changes, with 103 differentially expressed proteins (47 upregulated and 56 downregulated) (Supplemental Dataset S3, Supplemental Fig. S4, *C* and *D*). This is reflected in a robust separation between samples in the UMAP analysis. Deletion of both *hnox* and *nosP* was not merely additive, with 258 differentially expressed proteins identified (142 upregulated and 116 downregulated) (Supplemental Dataset S4). This observation suggests that H-NOX and NosP influence partially overlapping physiological processes despite being associated with distinct signaling pathways. A Venn diagram further illustrates this (Supplemental Fig. S4*E*), with little overlap between single and double mutations. Given the more substantial proteome alterations, we confined the remainder of our analyses to the Δ*xpc* strain to determine how the combined action of these two NO sensors influences bacterial physiology.

Prior to downstream analyses, we assessed the overall data quality for WT and Δ*xpc* samples (Fig. 2) by analyzing the distribution of protein intensities across biological replicates. The violin plots (Fig. 2*A*) showed that protein expression levels were distributed in a similar and symmetric way across WT and Δ*xpc* samples, confirming the high reproducibility of sample preparation and TMT quantification, as well as the absence of batch-related bias. The UMAP projection (Fig. 2*B*) demonstrated a clear segregation between WT and Δ*xpc* samples, which form two distinct clusters. The compact clustering of biological replicates within each genotype further supports the consistency and biological relevance of the observed differences. Consistent with the UMAP results, hierarchical clustering and heatmap analysis of protein intensities for all identified proteins (Supplemental Fig. S5) as well as for differentially expressed proteins (Fig. 2*C*) revealed two major branches corresponding to the WT and Δ*xpc* proteomes, underscoring the widespread impact of Δ*xpc* deletion on cellular protein expression. These analyses collectively validate the technical integrity of the dataset and illustrate that the disruption of H-NOX and NosP produces a distinctive and highly reproducible proteomic profile, indicative of a substantial remodeling of the cellular proteome.

**Fig. 2:**
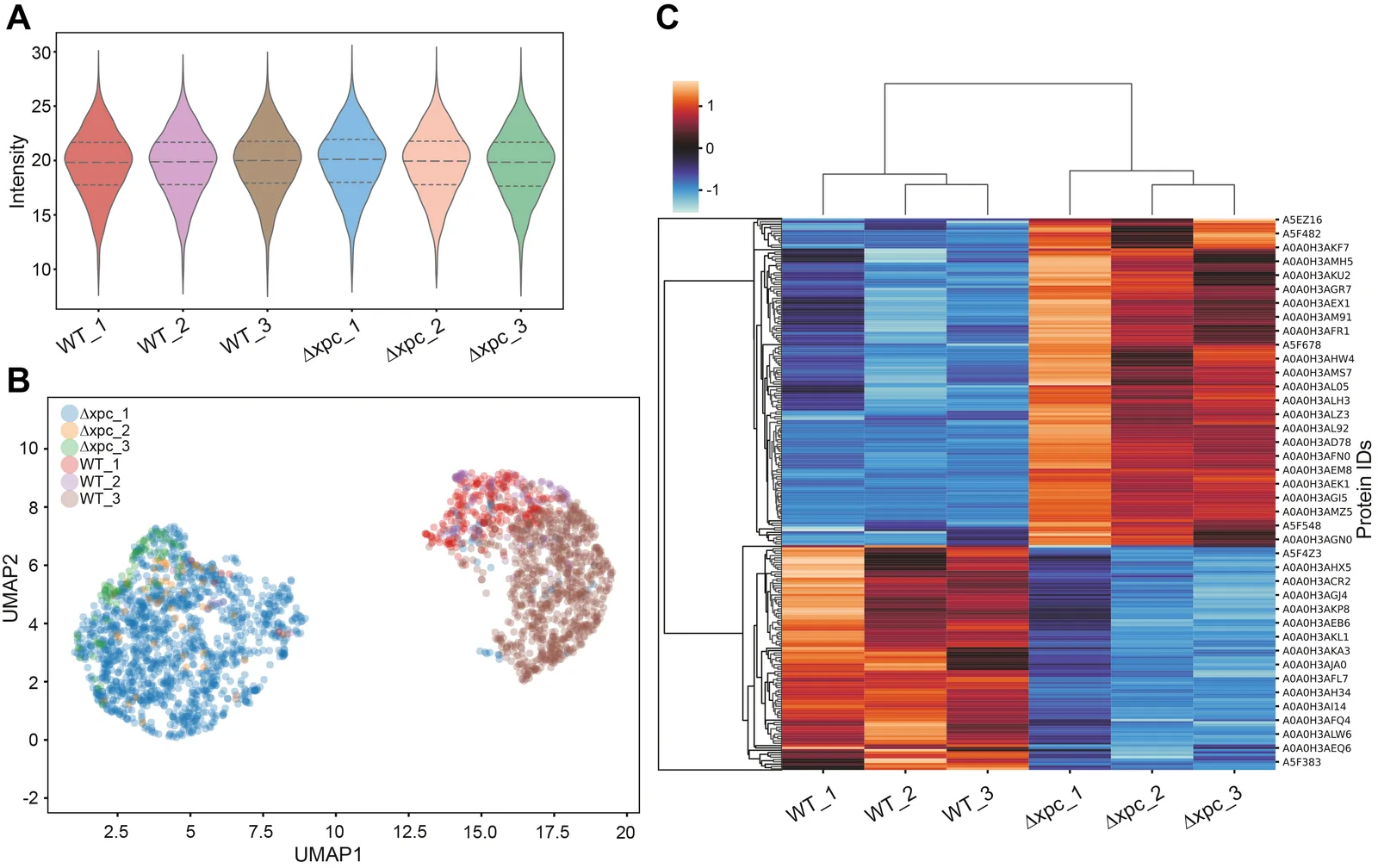
**Global proteomic comparison between WT and Δ*xpc* strains.***A*, violin plots showing the distribution of normalized protein intensities across biological replicates of WT and Δ*xpc* samples. *B*, UMAP analysis illustrate global proteome differences between WT and Δ*xpc* strains. *C*, hierarchical clustering and heatmap of differentially expressed proteins (n = 258). Each row represents a protein, and color intensity corresponds to z-scored expression values. Δ*xpc*, Δ*hnox*/*nosP*/*nadC*; UMAP, Uniform Manifold Approximation and Projection.

To elucidate the molecular pathways affected by the loss of H-NOX and NosP, next we performed differential proteomic analysis comparing the Δ*xpc* strain to the WT. The volcano plot (Fig. 3*A*) illustrates the global distribution of protein abundance changes, with a total of 258 proteins identified as differentially expressed (142 upregulated and 116 downregulated; *p* ≤ 0.05, |log_2_ FC| ≥ 0.58). To validate the proteomic findings, RT-qPCR analysis was performed for selected candidates (Fig. 3*B*). Consistent with the proteomic data, *vibA*, *viuA*, *vibB*, and *hutA* transcripts were all elevated in the Δ*xpc* relative to WT while the alkaline serine protease *ivaP* (VC0395_A2341) and VC0395_A0147 of the chitin utilization operon were downregulated. In each case, fold changes at the transcript level were substantially smaller than those observed at the protein level (Table 1). Posttranscriptional regulation, variable translation efficiency, and/or variable rates of RNA and protein degradation are all possible causes of this effect.

**Fig. 3:**
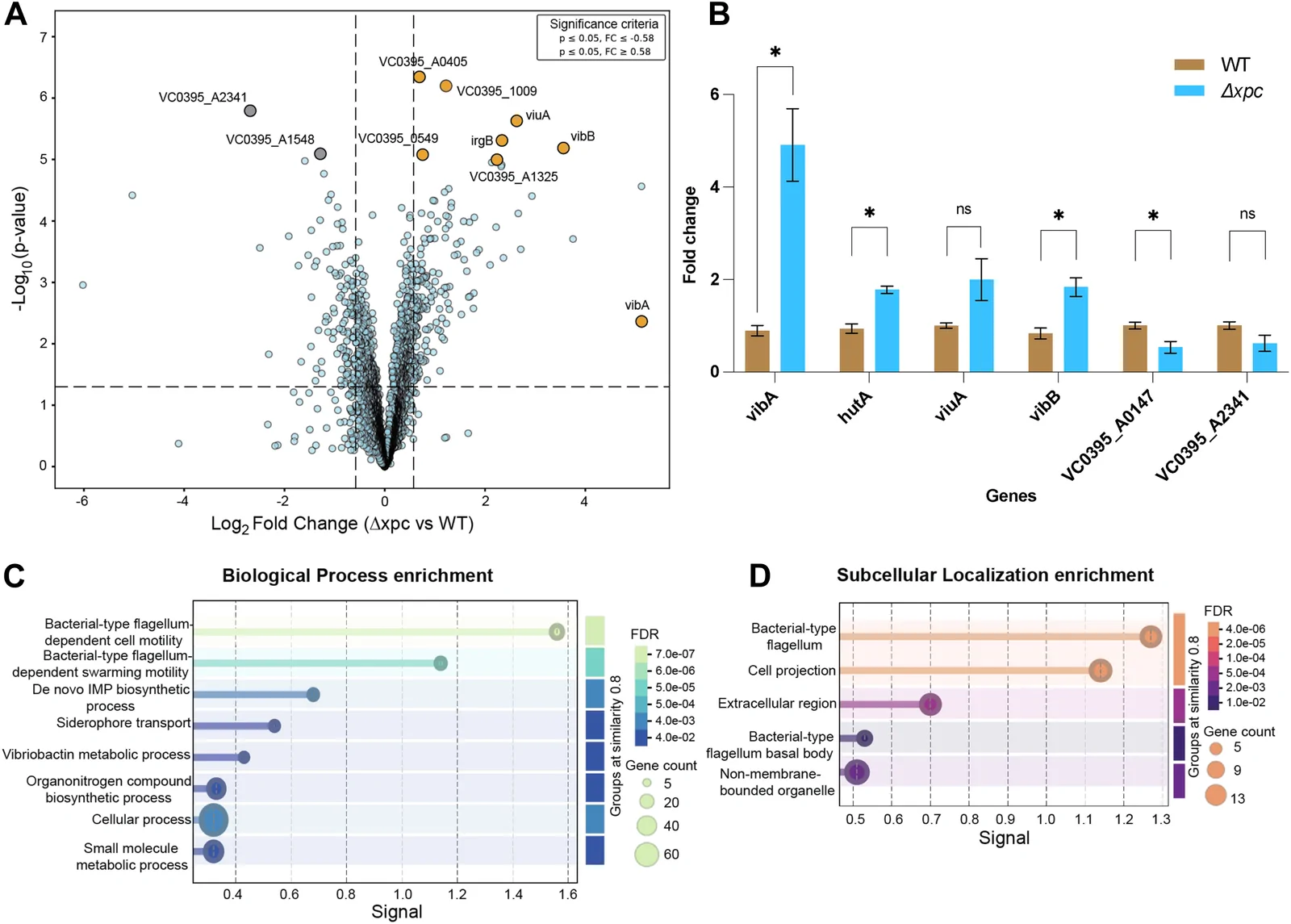
**Differential proteomic analysis in *Δxpc* mutant.***A*, volcano plot showing differential protein abundance between WT and *Δxpc* strains. Significance thresholds (*p* ≤ 0.05 and |log_2_ fold change| ≥ 0.58) are indicated by *dotted lines* with specific upregulated and downregulated proteins highlighted in *orange* and *gray*, respectively. *B*, RT-qPCR validation of selected candidates. Data represent mean ± SEM (n = 3); *p* < 0.05, Student’s *t* test. *C*, GO enrichment analysis (biological process) of upregulated proteins highlights enrichment of siderophore transport, vibriobactin biosynthetic process, and cellular metabolic pathways. *D*, subcellular localization enrichment reveals overrepresentation of extracellular and flagellar-associated proteins, consistent with alterations in surface architecture and motility. Δ*xpc*, Δ*hnox*/*nosP*/*nadC*; GO, Gene Ontology.

**Table 1:** Fold changes in Δ*xpc V. cholerae* compared to WT at the protein and transcript levels

Gene	Protein	Transcript
*vibA*	34.8	5.5
*hutA*	7.7	1.9
*viuA*	6.2	2.0
*vibB*	11.8	2.0
*VC0395_A0147*	0.36	0.54
*VC0395_A2341*	0.16	0.62

To gain functional insight into the broader biological impact of Δ*xpc* deletion, we performed Gene Ontology enrichment analysis based on the upregulated proteins. The biological process enrichment map (Fig. 3*C*) revealed strong enrichment for siderophore transport and vibriobactin metabolic processes, along with pathways related to organonitrogen compound biosynthesis, cellular metabolic processes, and flagellar-dependent motility. Furthermore, subcellular localization enrichment (Fig. 3*D*) highlighted an overrepresentation of proteins localized to the extracellular region, bacterial-type flagellum, and cell projection structures, consistent with altered surface architecture.

### Coordinated Regulation of Motility, Iron Acquisition, and Virulence Pathways in Δxpc

Several operons are conspicuous among the differentially expressed proteins in the Δ*xpc* strain (Fig. 4). Two operons encoding structural proteins of the polar flagellum (Fla and Flg proteins) had 16 proteins upregulated by 1.6 to 3.4-fold. The operon for biosynthesis of the siderophore vibriobactin (Vib proteins) was also upregulated, with VibA exhibiting the largest positive fold change of any protein at 34.8-fold. In contrast, the TCP operon was downregulated in the mutant strain at a level of 1.6 to 5.0-fold. Also downregulated was a chitin utilization operon regulated by the sensor kinase ChiS and encoding catabolic (VC0395_A0140-0144) and transport proteins (VC0395_A0145-0149) (46, 47, 48).

**Fig. 4:**
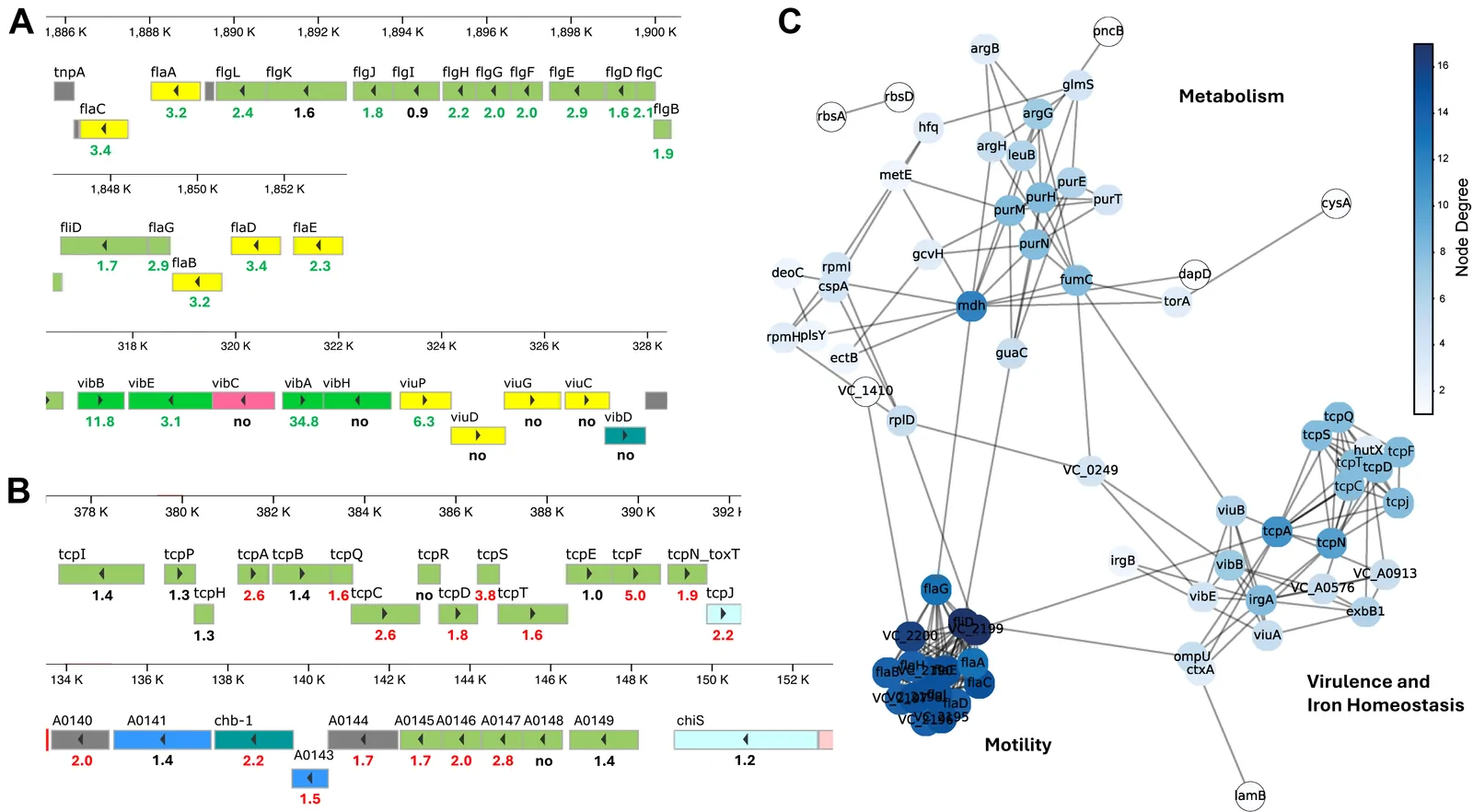
**Network interaction analysis of differentially regulated proteins in *Δxpc* mutant of *Vibrio cholerae*.** Operons that were significantly upregulated (*A*) or downregulated (*B*) in Δ*xpc versus* WT strains. Fold changes at the protein level are indicated by *green* (upregulated), *red* (downregulated), or *black* (below the threshold for fold-change and/or significance). Proteins not observed are indicated by “no”. *C*, PPI network constructed from significantly regulated proteins identified in the *Δxpc* strain relative to WT. The network was generated using STRING (confidence ≥0.7) and visualized in Python, with node degree indicating the number of interactions (color gradient = connectivity strength). Δ*xpc*, Δ*hnox*/*nosP*/*nadC*; PPI, protein–protein interaction.

To gain further insight into the biological interactions among the differentially expressed proteins, we generated a protein–protein interaction network using all significantly regulated proteins identified from the TMT-based proteomic dataset (Fig. 4*C*) with the node degree indicating the strength of the connections. Three clusters emerge from this analysis. The first labeled “Motility” includes flagellar proteins such as FlaA, FlaB, FlaC, FlaD, FlaE, FlgL, and FliD. The second labeled “Virulence and Iron Homeostasis” includes proteins of the vibriobactin and TCP operons as well as cholera toxin subunit A (CtxA), which is downregulated 3.3-fold in the Δ*xpc* strain. Proteins involved in iron acquisition are also observed in this cluster including heme transporter proteins HutA (outer membrane receptor, 7.7-fold upregulated) and HutX (cytosolic carrier protein, 3.6-fold upregulated), and the iron regulated virulence proteins IrgA (enterobactin outer membrane receptor, 5.0-fold upregulated) and IrgB (*irgA* transcriptional activator, 5.0-fold upregulated). Finally, the cluster labeled “Metabolism” is dominated by proteins of the *de novo* purine biosynthesis pathway (49) (PurE, PurH, PurM, PurN, and PurT, each downregulated 1.5–1.7-fold) and arginine biosynthesis pathway (ArgB, ArgG, and ArgH, each downregulated 1.7–2.8-fold). Other proteins identified in this cluster are involved in amino acid biosynthesis (MetB, 2.4-fold upregulated; LeuB, 1.6-fold upregulated), NAD^+^ biosynthesis (PncB, 1.6-fold upregulated), and the citric acid cycle (MDH, 1.6-fold upregulated; FumC, 1.9-fold upregulated).

Other differentially regulated proteins of interest were observed that did not fit into these clusters. The alkaline serine protease IvaP (VC0395_A2341) was downregulated 6.4-fold in the Δ*xpc* strain. IvaP is secreted in the human host (50) and can degrade the host protein intelectin (51), suggesting a role in host immune evasion or release from the intestinal epithelium. The MbaA protein (VC0395_A0233) was downregulated 2.1-fold in the Δ*xpc* strain. MbaA has both GGDEF and EAL domains, suggesting a role in c-di-GMP metabolism. Deletion of *mbaA* in *V. cholerae* El Tor results in alterations to biofilm morphology, suggesting a role in maintaining biofilm architecture (52). Finally, a predicted manganese superoxide dismutase (SodA, VC0395_A2266) was upregulated 4.7-fold in the Δ*xpc* strain. This is an intracellular enzyme important for the detoxification of superoxide radical (53).

### Motility Behavior of WT and Δxpc *V. cholerae*

To determine whether increased abundance of flagellar protein expression in the Δ*xpc* strain correlates with changes in spontaneous motility, we monitored swimming behavior in GFP-labeled WT and Δ*xpc* cells in LB media (Fig. 5). Both the WT and the Δ*xpc* strains were highly motile with orbital motility patterns. No changes were observed between strains in swimming speed, distance, or directional persistence. Thus, increased flagellar expression did not correlate with altered motility in motile *V. cholerae* cells.

**Fig. 5:**
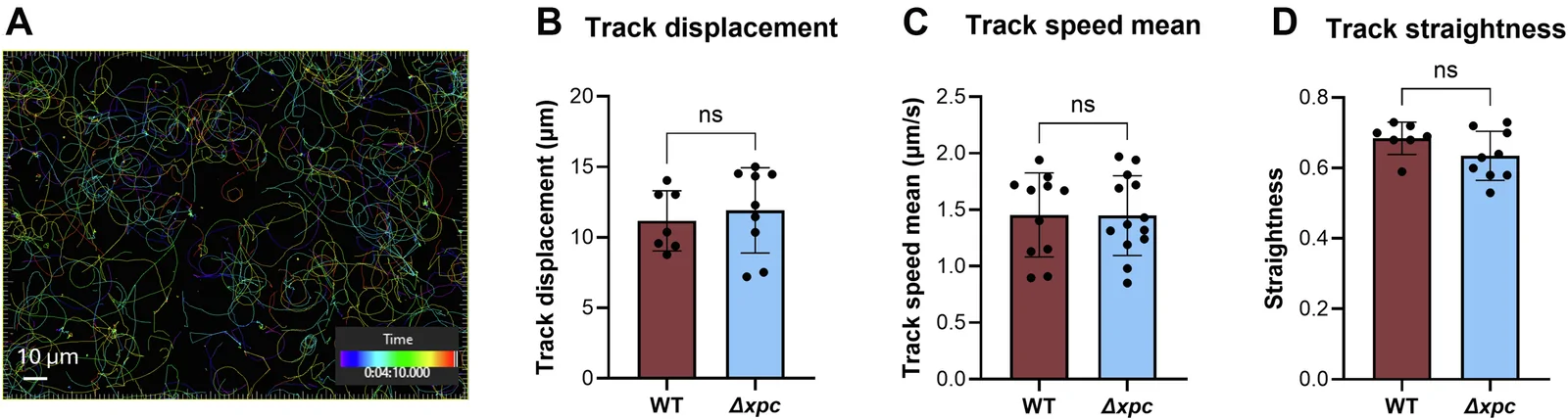
**Deletion of *Δxpc* does not change *Vibrio cholerae* swimming motility.** The indicated parameters were compared between WT and Δ*xpc* GFP-labeled cells using fluorescence microscopy. Motility features were analyzed using Imaris software. *A*, representative motility traces of WT *V. cholerae.* The scale bar represents 10 μm. *B*, track displacement, that is, total distance traveled; (*C*) mean track speed, (*D*) straightness, that is. track displacement/distance between start and end points. Data represent averages from n = 219 to 485 individual tracks per video from four independent experiments with 1 to 3 videos per strain and were analyzed using paired Student’s *t* test. Δ*xpc*, Δ*hnox*/*nosP*/*nadC*.

### Growth and Survival of Δxpc *V. cholerae*

Given the putative function of H-NOX and NosP as NO sensors and the observation that SodA (VC0395_A2266) is upregulated in the Δ*xpc* strain, we assessed the growth and survival of the Δ*xpc* and WT strains in increasing concentrations of the NO donor DETA-NONOate and superoxide source paraquat (Fig. 6). The Δ*xpc* strain showed appreciable resistance to high concentrations of paraquat while a modest survival defect was observed at high concentrations of DETA-NONOate.

**Fig. 6:**
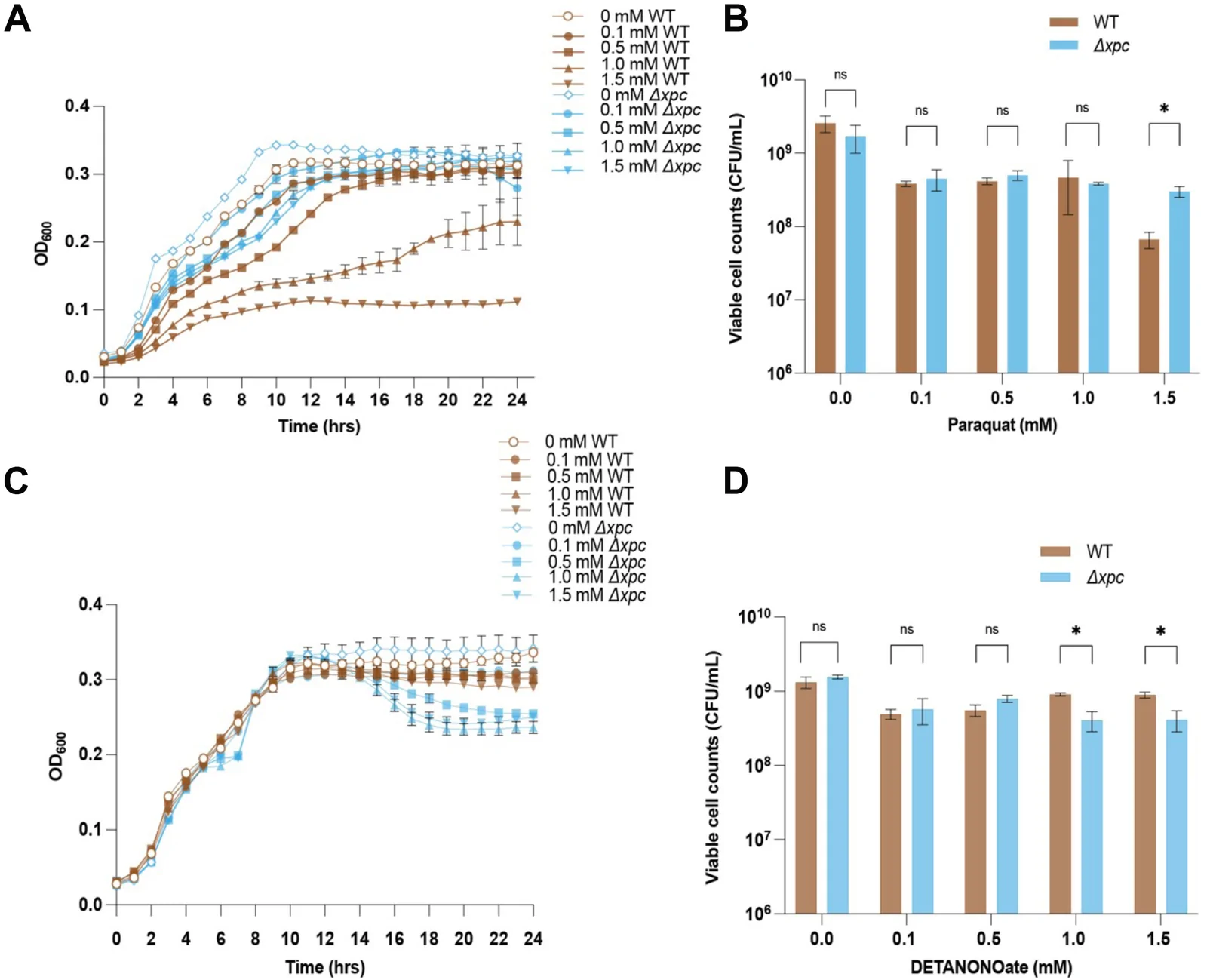
**Growth and survival of *V. cholerae* WT and *Δxpc*.** Growth and survival of *V. cholerae* WT (*brown*) and *Δxpc* (*blue*) strains in (*A*) LB growth medium supplemented with 0.1 to 1.5 mM of paraquat. *B*, viable cell counts (CFU/ml) following 24 h exposure to paraquat in LB medium. *C*, Growth in LB medium supplemented with 0.1 to 1.5 mM DETA-NONOate. *D*, viable cell counts (CFU/ml) following 24 h exposure to DETANONOate in LB medium. Results are from three replicate experiments with error bars reporting standard error of the mean (±SEM) (n = 3). Asterisks indicate a significant difference between treatments (∗*p* < 0.05) determined by an unpaired Student's *t* test. Δ*xpc*, Δ*hnox*/*nosP*/*nadC*; CFU, colony-forming unit; LB, Luria-Bertani.

### *V. cholerae* Δxpc Exhibits an Altered Transcriptional Response to Iron Limitation

Proteomic data revealed the upregulation of several iron acquisition systems in the Δ*xpc* strain compared to WT. To explore the effects of this, growth, iron accumulation, and transcription of iron acquisition genes were tested in MM with varying iron concentrations (Fig. 7). Growth at 30 μM and 3 μM added iron were virtually identical between strains, with only a very modest growth lag observed for the mutant. Both strains exhibited a severe growth defect when no iron was added to the media, precluding further analysis. Similarly, intracellular iron accumulation was highest in LB media and decreased with decreasing added iron in MM, but there was no significant difference between strains. In contrast, the transcriptional response to iron limitation was significantly different. As shown previously (Fig. 3*B*), *viuA*, *vibA*, *hutA*, and *vibB* were all transcribed at a higher level in Δ*xpc* than WT in LB media. These are known targets of iron-dependent transcriptional repression by the ferric uptake regulator (Fur) (54, 55), so their expression is expected to increase in response to iron limitation, as is observed for the WT. However, expression either does not change (*hutA* and *viuA*) or decreases with decreasing iron (*vibA* and *vibB*) in the Δ*xpc* strain (Fig. 7*C*).

**Fig. 7:**
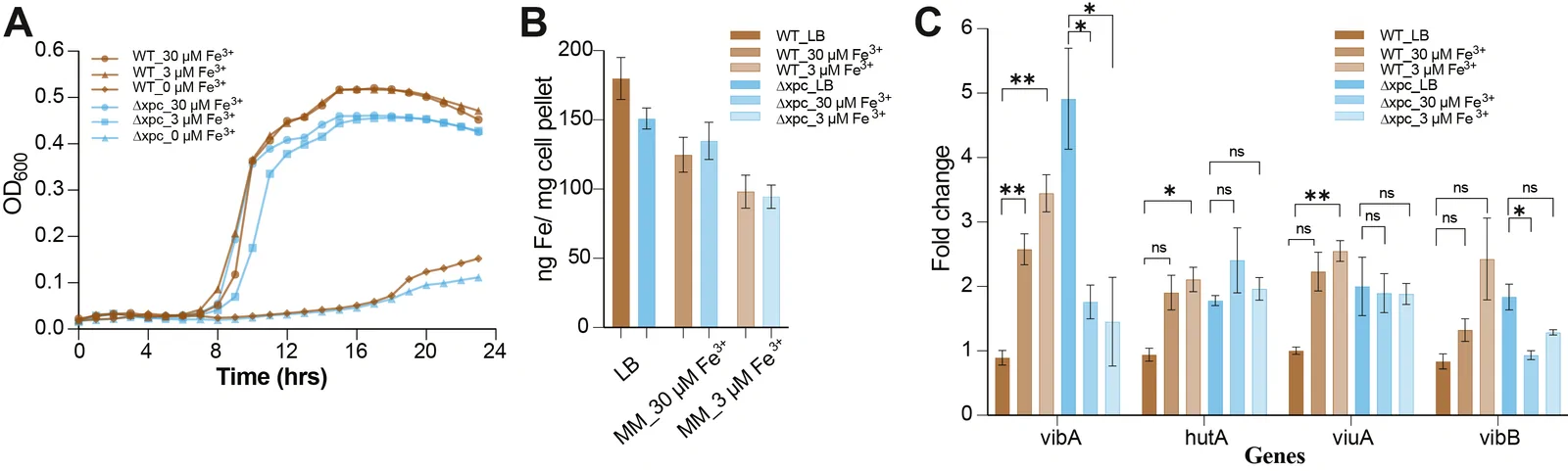
**The *Δxpc* mutant of *Vibrio cholerae* exhibits altered transcriptional response to iron limitation.***A*, growth of WT and *Δxpc* strains in MM supplemented with varying concentrations of Fe^3+^ (30 μM, 3 μM, and 0 μM). *B*, cellular iron content (ng Fe mg^−1^ cell pellet) in WT and Δ*xpc* strains grown in LB and MM containing 30 μM or 3 μM Fe^3+^. *C*, transcriptional response to iron limitation. RT-qPCR analysis of selected iron acquisition genes (*vibA*, *hutA*, *viuA*, and *vibB*) in WT and Δ*xpc* strains grown in LB and MM containing 30 μM or 3 μM Fe^3+^. Expression was normalized to WT grown in LB. Data represent mean ± SEM (n = 3); significance determined by Student’s *t* test (∗*p* < 0.05, ∗∗*p* < 0.01, ns = not significant). Δ*xpc*, Δ*hnox*/*nosP*/*nadC*; LB, Luria-Bertani; MM, minimal media; RT-qPCR, reverse transcription quantitative real-time polymerase chain reaction.

### Targeted Metabolomic Profiling Reveals Altered Metabolism in Δxpc

To examine the effect of the observed proteomic changes to metabolic enzymes, we carried out targeted metabolomic profiling of central carbon metabolites for WT and Δ*xpc* strains of *V. cholerae* during the mid-exponential growth phase (OD_600_ = 0.6) (Supplemental Dataset S5). The UMAP projection (Fig. 8*A*) demonstrated distinct segregation between the WT and Δ*xpc* metabolomes, characterized by tight clustering among biological replicates. This observation indicates a high level of reproducibility and highlights the distinct metabolic state associated with the Δ*xpc* strain. The volcano plot (Fig. 8*B*) revealed several metabolites that exhibited significant alterations in abundance (*p* ≤ 0.05, |log_2_ FC| ≥ 0.58). In particular, nucleotides and their precursors, pentose phosphate intermediates, and amino acids were differentially abundant between samples (Fig. 8*C*). The metabolite interaction network, accompanied by pathway clustering (Fig. 8*D*), demonstrated a coordinated reorganization of metabolic flux, particularly in glycolysis, the pentose phosphate pathway, and amino acid metabolism.

**Fig. 8:**
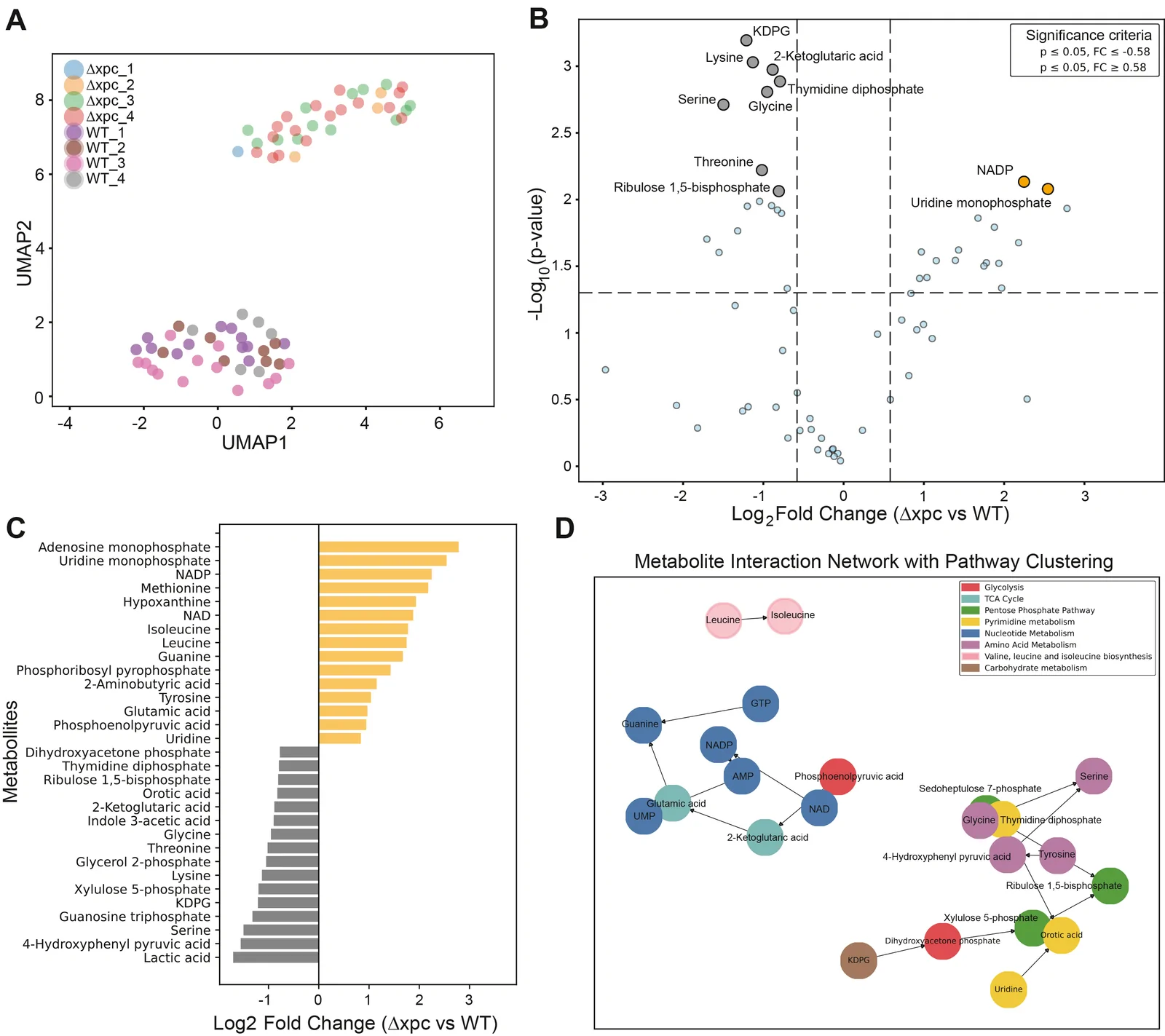
**Targeted ion-pairing LC–MS metabolomic profiling reveals metabolic reprogramming in *Δxpc* of *Vibrio cholerae*.***A*, UMAP projection showing clear separation between WT and Δ*xpc* metabolomes. *B*, volcano plot illustrating differentially abundant metabolites between WT and Δ*xpc* strains (*p* ≤ 0.05, |log_2_ fold change| ≥ 0.58). *C*, bar plot of log_2_ fold changes shows upregulated metabolites (*orange*) and downregulated metabolites (*gray*) in the *Δxpc* strain. *D*, metabolite interaction network with pathway clustering highlighting coordinated perturbations across metabolic pathways. *Arrows* represent pathway-level associations or shared biochemical steps rather than direct precursor–product conversions between individual metabolites. Δ*xpc*, Δ*hnox*/*nosP*/*nadC*; UMAP, Uniform Manifold Approximation and Projection.

### Phosphoproteomic Profiling Reveals Differential Phosphorylation of Regulatory Proteins in Δxpc

Given that both H-NOX and NosP are known to interact with kinases *in vitro* (21, 23, 56), we evaluated changes to the phosphoproteome using TMT-based phosphoproteomic analysis on WT and Δ*xpc* strains of *V. cholerae*. Peptides were generated from mid-exponential-phase cultures (OD_600_ ≈ 0.6) and labeled using a 10-plex TMT reagent and enriched for phosphopeptides with TiO_2_ and Fe-NTA prior to LC–MS^3^ analysis on an Orbitrap Eclipse Tribrid mass spectrometer. A total of 182 confidently localized phosphosites were identified, corresponding to 173 class A sites (localization probability ≥0.70) and 9 class B sites (localization probability <0.70) (Fig. 9*A*, Supplemental Dataset S6). Among these, serine phosphorylation predominated, followed by threonine, tyrosine, aspartate, and histidine residues (Fig. 9*B*), consistent with phosphosite identification in other bacterial species and likely underrepresenting phosphohistidine sites due to the acid lability of this modification (57).

**Fig. 9:**
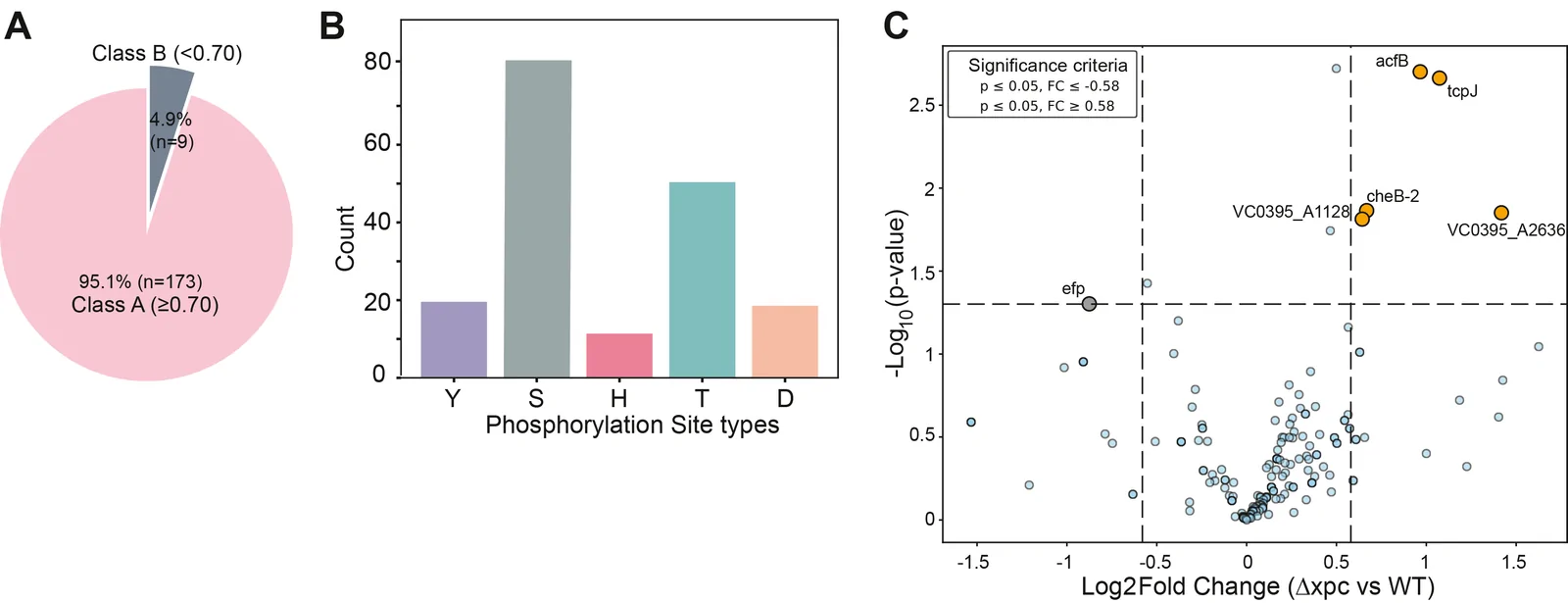
**Phosphoproteomic profiling reveals differential phosphorylation of regulatory proteins in Δ*xpc* of *Vibrio cholerae*.***A*, phosphosite localization confidence. Pie chart shows the distribution of class A (localization probability ≥0.70) and class B (<0.70) phosphosites identified in the dataset. *B*, bar plot showing the frequency of phosphorylated residues; serine (S) was the most abundant, followed by threonine (T), tyrosine (Y), aspartate (D), and histidine (H). *C*, volcano plot depicting log_2_ fold change (Δ*xpc versus* WT) against −log_10_*p*-value. Significantly upregulated phosphoproteins (*p* ≤ 0.05, |log_2_ FC| ≥ 0.58) are highlighted in *orange*. Δ*xpc*, Δ*hnox*/*nosP*/*nadC*.

The volcano plot (Fig. 9*C*) revealed several significantly upregulated phosphoproteins in the Δ*xpc* strain and only one downregulated. Several other phosphoproteins were only observed in the Δ*xpc* or WT (Table 2). VieS is known to autophosphorylate on His 1142 with phosphotransfer to D1018 (63), but the Ser 654 site identified here has not been previously described. A representative MS/MS spectrum of VieS phosphorylated peptide is shown in Supplementary Figure S6. CheB-2 is also known to be phosphorylated 61, 62, although the exact residue has not been identified. The remaining phosphoproteins listed in Table 2 and most of those in Supplementary Dataset S6 have not been previously observed to our knowledge.

**Table 2:** Phosphopeptides with differential abundance in Δ*xpc* compared to WT

Gene locus	Gene name	Function	Site	Phosphopeptide	Log_2_ ratio (Δxpc *versus* WT)
VC0395_A0364	tcpJ	Protease involved in pilin (TcpA) secretion (58)	Tyr 39	NHFF**Y**(ph)DIDSNR	1.07
VC0395_A0365	acfB	Methyl accepting chemotaxis transducer (59)	Ser 609	ISQQAY**S**(ph)QK	0.97
VC0395_A2636	rfbT (aka webT or tsfB)	Methyltransferase involved in seroconversion (60)	Thr 15	**T**(ph)ELDAIQSK	1.42
VC0395_A1650	cheB-2	Methyltransferase involved in chemotaxis (61, 62)	Ser 155	PAPTTSIAASS**S**(ph)LSQER	0.67
VC0395_A1128	n/a	Sensor histidine kinase (inferred)	His 550	A**H**(ph)NSVEVNR	0.64
VC0395_A2234	efp	Translation elongation factor (inferred)	Thr 3	AT(ph)VSTNEFK	−0.88
VC0395_A1259	vieS	Histidine kinase that phosphorylates VieA (63)	Ser 654	N**S**(ph)ESQLLLTNAISSAER	WT only
VC0395_A2310	pckA	Phosphoenolpyruvate carboxykinase (inferred)	Thr 79	DD**T**(ph)TRDTLWWTSDK	Δ*xpc* only
VC0395_A0349	tpx	Thiol-specific peroxidase (inferred)	Thr 72	VLQNALM**T**(ph)R	Δ*xpc* only
VC0395_0456	cqsA	Autoinducer synthase (64)	Ser 313	**S**(ph)ESQIIGLETGDER	Δ*xpc* only
VC0395_0456	cqsA	Autoinducer synthase (64)	Ser 315	SE**S**(ph)QIIGLETGDER	Δ*xpc* only

## Discussion

In the current study, integrated-omics approaches were utilized to evaluate the molecular impacts of the deletion of the NO sensors *hnoX* and *nosP* in *V. cholerae*. Total proteomics demonstrated that virulence factors such as TCP and CtxA were downregulated while flagellar proteins were upregulated in Δ*xpc V.cholerae*. Phosphoproteomics revealed changes in the phosphorylation of proteins involved in chemotaxis, c-di-GMP metabolism, and quorum sensing, further implicating H-NOX/NosP signaling in the transition between sessile and motile lifestyles. Vibriobactin biosynthesis and utilization proteins and Mn-dependent SodA were also upregulated even in replete iron conditions, suggesting an intersection between H-NOX/NosP and Fur signaling. SodA upregulation correlated with increased resistance to paraquat in the mutant strain. Finally, changes were observed in the abundance of metabolic proteins involved in purine and amino acid biosynthesis that correlated to changes in some central carbon metabolites. Taken together, the results highlight NO-independent signaling roles for H-NOX and NosP that help mediate the transitions between sessile and motile lifestyles that are critical for *V. cholerae* pathogenesis, persistence, and transmission.

### Regulation of Flagellar Biosynthesis and Virulence Factors

During the late stage of *V. cholerae* infection, a subset of bacterial cells initiates a mucosal escape response to detach from the intestinal epithelium and migrate to the lumen. This is characterized by upregulation of flagellar biosynthesis and chemotaxis genes and a downregulation of virulence factors including TcpA and CtxA (65). This is a strikingly similar situation to what is observed in Δ*xpc V*. *cholerae*, suggesting that H-NOX and NosP may suppress this response. To that end, it is compelling that the mucosal escape response requires HapR, the end point of the Lux quorum sensing circuit in which NosP participates (23) (Fig. 1). A role for H-NOX/NosP in regulating the escape response is also consistent with the downregulation in the mutant strain of the serine protease IvaP, which may facilitate release from the epithelial surface by cleaving host intelectin (50, 51). Conversely, late-stage infection induces chitin utilization genes of the ChiS regulon as well as the C4-dicarboxylate transport transcriptional regulator (VC0395_A1516) (66), which were downregulated 1.5 to 2.8-fold and 2.3-fold, respectively, in the Δ*xpc* mutant. Increased expression of these proteins is posited to prime *V. cholerae* to make use of new carbon sources upon dissemination to the environment.

We also observed upregulation of flagellar proteins in the Δ*xpc* mutant relative to WT. However, these did not elicit an increase in individual cell motility as determined by microscopy. This observation might be rationalized by comparison to a very recent report where NO exposure led to *V. cholerae* biofilm dispersal and detachment from host cells through upregulated transcription of flagellar genes (20) but did not enhance motility. Rather, NO promoted the biosynthesis of flagella in biofilm-associated cells, which are largely aflagellated (67), driving a transition from a sessile to motile lifestyle that may be important for infection, dispersal, or both. Individual deletions of nitrosative stress sensors *norR* (68), *aphB* (69), or *hnox*/*hnoK* had no effect on NO-induced biofilm dispersal, but the triple mutation attenuated the response, indicating redundant roles for these sensors, similar to what we observe for *hnox* and *nosP*, as well as the existence of other required mechanisms. The role of *nosP* was not evaluated, but the current work suggests that it may also contribute to NO-mediated dispersal.

The observation that both NO exposure and deletion of *hnox* and *nosP* result in upregulation in flagellin production suggest that these proteins have an NO-independent function in repressing flagellar protein expression that is relieved upon NO binding. In the current work, exogenous NO was not added and *V. cholerae* lacks a functional nitrite reductase (70) that could produce NO endogenously. Notably, formation of a complex between *V. cholerae* H-NOX and HnoK is not dependent on NO (56), and *Shewanella oneidensis* H-NOX in the unligated Fe(II) state actually stimulated HnoK autophosphorylation activity more than 2-fold compared to HnoK alone (71). These *in vitro* studies provide a mechanism whereby H-NOX, and by inference NosP, may exhibit NO-independent signaling functions consistent with what is observed here.

Another NO-independent function of H-NOX/NosP appears to be the activation of virulence factors including TCP proteins and CtxA, which are downregulated in the Δ*xpc* mutant. Virulence genes are under the direct control of the transcriptional activator ToxT (aka TcpN), which is itself activated in a complex process by transmembrane transcription factors ToxRS and TcpPH along with cytoplasmic regulator AphAB (72, 73, 74, 75, 76, 77, 78). ToxT expression is also induced upon contact with host cells through induction of the PDE enzyme VieA, presumably through the reduction of c-di-GMP levels (79). VieA activates its own expression (80) upon phosphorylation by the sensor His kinase VieS, resulting in diminished levels of c-di-GMP, which promote motility and inhibit biofilm formation (63). Our proteome data show no differential expression of VieA between WT and Δ*xpc* strains. However, noncanonical phosphorylation of VieS at a Ser residue was observed only in WT. The function of this modification is unknown, but it provides a loose connection between virulence gene regulation by H-NOX/NosP and the VieSA system that may be worth further investigation.

### Iron Homeostasis, Fur Regulation, and Oxidative Stress

Several of the most significantly upregulated proteins in Δ*xpc V. cholerae* are associated with iron acquisition and transport. These include proteins involved in vibriobactin biosynthesis (VibB, VibE, VibA) and transport (ViuP), heme uptake (HutA and HutX), and enterobactin utilization (IrgA and IrgB). All are negatively regulated by Fur and iron (55). Curiously, intracellular iron accumulation was not affected by the Δ*xpc* mutation despite this upregulation. Rather, Fur targets appeared to be constitutively expressed in the mutant irrespective of iron concentration in the media, suggesting some disruption of Fur function. Interestingly, *tcp* genes are also positively regulated by Fur independently of iron, and a Δ*fur* mutant was deficient in colonization of an infant mouse model (55). Thus, crosstalk between H-NOX/NosP and Fur signaling may provide a mechanism for the differential regulation of both iron acquisition genes and *tcp* genes observed in this work.

The fumarase FumC and SodA are also known Fur targets (55), and both were upregulated in the Δ*xpc* strain. SodA expression correlated with paraquat resistance in Δ*xpc V. cholerae*, and previous studies have demonstrated a function for SodA in paraquat resistance in several bacterial species including the human pathogens *Klebsiella pneumoniae* (81) and *Acinetobacter baumannii* (82). *V. cholerae* H-NOX can act as a sensor of other oxidants such as hypochlorite, diamide, and hydrogen peroxide *in vitro* (15, 21). However, differential expression of SodA protein was not detected for the Δ*hnox* single mutant (Supplemental Dataset S2), ruling out an independent role for H-NOX in superoxide-dependent regulation of *sodA*.

### Metabolic Changes

A substantial proportion of differentially regulated proteins in Δ*xpc V. cholerae* are involved in metabolism. These included proteins involved in *de novo* purine biosynthesis, amino acid and NAD^+^ biosynthesis, and citric acid cycle enzymes, among others. These changes correlated with changes in the relative abundance of central carbon metabolites including amino acids, nucleic acids, pentose phosphate and glycolytic intermediates. Some correlations are intuitive (*e*.*g*. increased Met coupled to increased MetB) while others are not (*e*.*g*. increased hypoxanthine and guanine coupled to decreased purine biosynthesis enzymes), underscoring the complexity of dynamic metabolic pathway regulation that is not well captured by single-timepoint quantitation of metabolic intermediates. Furthermore, we cannot rule out the possibility that the succinate transporter *nadC* deletion in Δ*xpc V. cholerae* (see Experimental Procedures) may be responsible for some altered metabolite levels, although the redundancy of this gene in *V. cholerae* and the unimpaired growth of the Δ*xpc* strain on succinate argue against a major role. In any case, it is certain that metabolic reprograming is essential for survival in the various environments that *V. cholerae* inhabits, and this data suggest a role for H-NOX/NosP signaling in this process. It is interesting to note that multiple genes involved in purine and amino acid metabolism have been identified as important for infection (83).

### Protein Phosphorylation

Phosphorylation events are prominent in H-NOX and NosP signaling, prompting us to undertake a comprehensive analysis of *V. cholerae* protein phosphorylation. A total of 182 phosphosites across 147 proteins were identified. To our knowledge this is the first such dataset for this species, and it is hoped that it will be a valuable resource for other researchers.

Only 10 proteins were differentially phosphorylated between WT and Δ*xpc V. cholerae* (Table 2)*.* Among these, two (AcfB and CheB-2) are involved in chemotaxis (59, 61, 62) and are more highly phosphorylated in the mutant strain. TcpJ is also more highly phosphorylated in the mutant. TcpJ is a protease responsible for cleaving the TcpA leader sequence to yield the mature form competent for secretion and formation of the TCP (58). To our knowledge, phosphorylation of this protein has not been previously observed. Based on repressed TcpA in the mutant, we might conjecture that phosphorylation of TcpJ would be inhibitory, although this will require further genetic and/or biochemical experiments to confirm.

CqsA was phosphorylated at two sites and only detected in the mutant strain. CqsA is essential for the synthesis of the quorum sensing autoinducer CAI-1, which negatively regulates biofilm formation and enhances colonization efficiency (64, 84, 85). Crystal structures exist for *V. cholerae* CqsA (86, 87) and show that the phosphorylated Ser residues are surface exposed and in contact with a loop near the active site, making it likely that phosphorylation would influence enzyme activity. To our knowledge, phosphorylation of an autoinducer synthase has never before been observed as a regulatory mechanism. CqsA, with available structures and *in vitro* activity assays, provides a tractable system to test this mechanism. TpxA and Pck, annotated as a thiol-specific peroxidase and phosphoenolpyruvate carboxykinase, respectively, are two other enzymes phosphorylated only in the mutant strain with possible roles in oxidative stress response (88) and carbon metabolism (89). A final phosphoprotein of interest identified here is a putative sensor histidine kinase (VC0395_A1128) that was more phosphorylated in the WT. This gene is adjacent to that of a response regulator (VC0395_A1129), suggesting that it functions as part of a two-component regulatory system. Although apparently uncharacterized in *V. cholerae*, this putative two-component regulatory system is homologous to the FlgS/FlgR system in *Campylobacter jejuni* that regulates flagellar gene transcription (90). Thus, it may represent yet another sensory system influencing motility in *V. cholerae*.

## Conclusions

Here we show that H-NOX/NosP signaling suppresses flagellar biosynthesis and some iron acquisition systems while promoting expression of virulence factors and purine biosynthesis genes. Crosstalk with Fur signaling provides a possible mechanism for many of these expression changes. Perhaps most compelling is that virtually all the virulence genes and purine biosynthesis genes identified in this study were important fitness factors during infection of an infant rabbit model while the flagellar genes were important for survival upon dissemination into the aquatic environment (83). Thus, it seems likely that H-NOX and NosP are important for the transition to host colonization and infection and/or maintenance of the infectious phenotype. While *in vitro* data (21, 22, 23) and modest sensitivity to NO observed in the double mutant strain indicates that H-NOX and NosP likely respond to NO, the protein expression changes identified here are independent of NO. However, biofilm dispersal through upregulation of flagellar genes upon NO exposure suggests that it may relieve their repression by H-NOX/NosP, signaling transition to a motile phenotype during early-stage infection or to facilitate exit from the host (20). Phosphoproteome data presented here reinforces the prominence of virulence factors and motility proteins in H-NOX/NosP signaling, providing opportunities to investigate new targets involved in *V. cholerae* pathogenesis.

## Data Availability

Mass spectrometry data have been deposited to the ProteomeXchange Consortium *via* the jPOSTrepo partner repository and can be accessed from https://repository.jpostdb.org/entry/JPST004424.0 (https://repository.jpostdb.org/preview/177269172469960d5045511, access key 4609). Annotated spectra can be viewed using the following link https://msviewer.ucsf.edu/prospector/cgi-bin/mssearch.cgi?report_title=MS-Viewer&search_key=efwgarpbp9&search_name=msviewer.

## Supplemental Information

This article contains supplemental information (32, 34, 91).

## Conflict of Interest

The authors declare no competing interests.

## Supplemental data

Supplemental Data Set 1

Supplemental Data Set 2

Supplemental Data Set 3

Supplemental Data Set 4

Supplemental Data Set 5

Supplemental Data Set 6
