# Supplementary material for: H-NOX and NosP Regulate Flagellar Protein and Virulence Factor Production in Vibrio Cholerae
Source: Mol Cell Proteomics. 2026 Sep 3;25(10):101654. doi: 10.1016/j.mcpro.2026.101654 (PMC13654575; doi:10.1016/j.mcpro.2026.101654)
Supplement: Supplementary file 1 [file mmc7.docx]

**Supporting Information**

**H-NOX and NosP Regulate Flagellar Protein and Virulence Factor Production in *Vibrio cholerae***

**Thathmi Wathsara Herath Pathiranage^1^, Md. Shariful Islam^1,2^, Cedar Stellon^3^, Michelle D. Cherne^3,4^, Daniel Valencia^1^, Diane Bimczok^3,5^, and Erik T. Yukl^1*^**

^1^Department of Chemistry and Biochemistry, New Mexico State University, Las Cruces, NM 88003.

^2^Current Affiliation: Department of Natural Sciences, University of Maryland Eastern Shore, Princess Anne, MD 21853

^3^Department of Microbiology and Cell Biology, Montana State University, Bozeman, MT 59717.

^4^Current affiliation: Department of Biomedical Engineering and Science, Florida Institute of Technology, Melbourne, FL 32901

^5^Center for Biofilm Engineering, Montana State University, Bozeman, MT 59717

^*^Correspondence: Tel. 575 646 3176; E-mail: [etyukl@nmsu.edu](mailto:wilmo004@umn.edu)

**Supplementary Table 1: Bacterial strains and plasmids used in this study**

| Strain or Plasmid | Description | Reference or Source |
| --- | --- | --- |
| Strains |  |  |
| *Vibrio cholerae* O395 | Classical biotype, Str^r^ | F. Yildiz |
| *Vibrio cholerae* O395:*ΔhnoX* | *hnoX* deletion mutant | This study |
| *Vibrio cholerae* O395:*ΔnosP* | *nosP* deletion mutant | This study |
| *Vibrio cholerae* O395:*ΔhnoX/ΔnosP* | *hnoX/nosP* deletion mutant also containing a 14bp deletion in *nadC* (VCO395_0108) | This study |
| *Vibrio cholerae* O395*::Tn7gfp* | Classic biotype with constitutive GFP reporter integrated via Tn7 | This study |
| *Vibrio cholerae* O395 *ΔhnoX::Tn7gfp* | *hnoX* deletion mutant with constitutive GFP reporter integrated via Tn7 | This study |
| *Vibrio cholerae* O395 *ΔnosP::Tn7gfp* | *nosP* deletion mutant with constitutive GFP reporter integrated via Tn7 | This study |
| *Vibrio cholerae* O395 *ΔhnoX/ΔnosP::Tn7gfp* | *hnoX/nosP* deletion mutant with constitutive GFP reporter integrated via Tn7 | This study |
| *Vibrio cholerae* O395 *ΔhnoX/ΔnosP::pBAD-nadC* | *hnoX/nosP* deletion mutant with arabinose inducible pBAD18:kan plasmid carrying *VCO395_0108*, transporter NadC family | This study |
| *E.coli DH5α* | Cloning strain |  |
| *E.coli SM10 λpir* | Conjugation strain | F. Yildiz |
| Plasmids |  |  |
| pUX-BF13 | oriR6K helper plasmid providing Tn7 transposition function in *trans*; Ap^r^ | (1) |
| pTn7-gfp | Vector for Tn7 insertion of *gfp*; Gm^r^ Ap^r^ | F. Yildiz |
| pGP704-SacB | Suicide vector pGP704 carrying *sacB*; Ap^r^ | (2) |
| pGP704-SacB::*ΔhnoX* | Suicide vector pGP704**-**SacB for *hnoX* deletion | This study |
| pGP704-SacB::*ΔnosP* | Suicide vector pGP704**-**SacB for *nosP* deletion | This study |
| pGP704-SacB::*ΔnadC* | Suicide vector pGP704**-**SacB for *nadC* deletion | This study |
| pBAD18-kan | Arabinose-inducible expression vector; Kn^r^ | (3) |
| pBAD18-kan::*hnoX* | pBAD18-kan carrying *hnoX* | This study |
| pBAD18-kan::*nosP* | pBAD18-kan carrying *nosP* | This study |
| pBAD18-kan::*nadC* | pBAD18-kan carrying *nadC* (VCO395_0108) | This study |

**Supplementary Table 2: Primers used in this study**

| Construct | Primer | Sequence (5' to 3') |
| --- | --- | --- |
| pGP704:SacB::*ΔnosP* |  |  |
| 5' insert | FWD | TCT GGA TCC ACG AAG CTT CC ATC TAA ACT GTT AAT TCT TGTTGC TGA |
|  | REV | ACT TTC TCT GTG TAT TCA GAA TCC ATT T TAT TTA TAC CTG ATA TAT CGGAGTGTTT |
| 3' insert | FWD | AAA CAC TCC GAT ATA TCA GGTA TAA AT A AAA TGG ATT CTG AAT ACA CAG AGA AAGT |
|  | REV | ATC TAG AAC CGG TGA CGT CAC AAGTTGGACTTAGATTGGTTAGCTTG |
| pGP704:SacB::*ΔhnoX* |  |  |
| 5' insert | FWD | TCT GGA TCC ACG AAG CTT CC GGT CTA TCG GTT TTC CAA TGG TGG |
|  | REV | GCG TTG ATA TTG CGC TTC GGC AC TCA AGT GTT GTA GAG ATA AGG AAA AGC C |
| 3' insert | FWD | GGC TTT TCC TTA TCT CTA CAA CAC TTG A GTG CCG AAG CGC AAT ATC AAC GC |
|  | REV | ATC TAG AAC CGG TGA CGT CAC ATC AGC ACT TGC TGT ATT TCA GGGG C |
| pGP704:SacB::*ΔnadC* | n/a | ~650 bp 5’ and 3’ regions flanking *nadC* were synthesized as a fused construct by Twist Bioscience and cloned into pGP704:SacB at an NcoI restriction site. |
| pBAD::kan *hnoX* | FWD | GCT CAG CTA GCA TCT CTA CAA CAC TTG AAT GAA AAC AAG CGC A |
|  | REV | ACT GCT CTA GAA TGC GCT TCG GCA CTC ATG ATG GCA AAA A |
| pBAD::kan *nosP* | FWD | CTG CGC TAG CCG ATA TAT CAG GTA TAA ATA GTG TCA TCT CAC GTA TAC AA |
|  | REV | CTA CTC TAG AGT GTA TTC AGA ATC CAT TTT CAT GCC TCA TAA AAT ACA AC |
| pBAD::kan *nadC* | FWD | GCT CGG TAC CCG GGG ATC CTA GGA GGA ATT |
|  | REV | CTT GCA TGC CTG CAG GTC GAC T |
| RT-PCR Genes | **Primer** | **Sequence (5' to 3')** |
| *gfp* | FWD | ATG CGT AAA GGA GAA GAA CTT TT |
|  | REV | TCA GCT AAT TAA GCT TAA TTT GTA TAG T |
| *vibA,* VC0395_A0303 | FWD | CGC ATT ACA CAT GCT GGT TAA G |
|  | REV | ACC ATA CTG CTC AGT CCA AAG |
| *hutA,* VC0395_0519 | FWD | TGC GGC ATC ATC ACT ACA A |
|  | REV | TCG CAT AGC CAC CCA TAT TAC |
| *viuA,* VC0395_A1803 | FWD | CGG TCG TTC GCT GAC TTA T |
|  | REV | GCC CTT GAA TGT AGC TCT GT |
| *vibB,* VC0395_A0300 | FWD | GTG ATG TTC AGC TCA CCA AAT G |
|  | REV | CGC ATA GAC ACC CGT AAT GAT |
| *nadC,* VC0395_0108 | FWD | CTT CAT TGC GGT GCT TT |
|  | REV | ATC GAG TTG GCA AAG TTA TT |
| *ivaP,* VC0395_A2341 | FWD | CAA ATG CGG CTC AAG ATA ACG |
|  | REV | TAA CGC CAC TGC CAT CAT AG |
| VC0395_A0147 | FWD | CGT GAG AAA GAG TTT GTG AAA G |
|  | REV | GTA CAT CAC CGA ACC GAT AAA |
| GAPDH, VC0395_A0587 | FWD | GTG ATT GAG GCT ACG GGT AAG |
|  | REV | CTT CTT TGA CAG GTG CTG AAA C |

**Supplementary Figure 1.** (A) Expression of *nadC*. Primers were designed to amplify ~100 bp of *nadC* with the forward primer annealing to the missing 14 bp region missing in the Δ*xpc* strain using the following templates. Lane 1, 1 kb ladder; 2, WT gDNA; 3, WT 50 ng cDNA; 4, WT 10 ng cDNA; 5, Δ*xpc* 50 ng cDNA; 6, Δ*xpc* 10 ng cDNA; 7, Δ*xpc*:pBAD-*nadC* 50 ng cDNA; 8, Δ*xpc*:pBAD-*nadC* 10 ng cDNA. (B) Growth of *V. cholerae* WT, *Δxpc,* and *Δxpc*:pBAD-*nadC* in LB media. Arabinose was added to 0.1% w/v to the Δ*xpc:pBAD-nadC* to induce *nadC* expression. Results are from three replicate experiments with error bars reporting standard error of the mean (±SEM) (n=3).

**Supplementary Figure 2.** Growth of *V. cholerae* WT (brown) and Δ*xpc* (blue) in minimal media containing 10 (circles) or 50 mM (squares) succinate as the sole carbon source. Results are from three replicate experiments with error bars reporting standard error of the mean (±SEM) (n=3).

**Figure S3: RT-qPCR of selected candidates in Δ*nadC* vs. WT.** Transcript levels of *vibA*, *hutA, A0147,* and *A2341* in the Δ*nadC* strain relative to WT. Data represent mean ± SEM (n = 3); *p* < 0.05, Student’s *t*-test.

**Figure S4: Global proteomic comparison between WT and Δ*hnox* (A and B) or Δ*nosP* (C and D).** (A and C) Uniform Manifold Approximation and Projection (UMAP) analysis illustrating global proteome differences between WT and mutant strains. (B and D) Volcano plot showing differential protein abundance between WT and mutant strains. Selected proteins meeting the significance threshold (*p* ≤ 0.05 and |log₂ fold change| ≥ 0.58) are highlighted in orange and grey. (E) Venn diagram showing the overlap between differentially expressed proteins identified in all strains in this study.

**Figure S5: Hierarchical clustering and heatmap of all quantified proteins (n = 2,812).** Each row represents a protein, and color intensity corresponds to z-scored expression values.

**Figure S6: MS/MS spectrum of VieS phosphorylated peptide.** Blue and red lines indicate y- and b-ions, respectively.

**References**

1.	Bao, Y., Lies, D. P., Fu, H., and Roberts, G. P. (1991) An improved Tn7-based system for the single-copy insertion of cloned genes into chromosomes of gram-negative bacteria *Gene* **109**, 167-168

2.	Miller, V. L., and Mekalanos, J. J. (1988) A novel suicide vector and its use in construction of insertion mutations: osmoregulation of outer membrane proteins and virulence determinants in Vibrio cholerae requires toxR *J Bacteriol* **170**, 2575-2583

3.	Guzman, L. M., Belin, D., Carson, M. J., and Beckwith, J. (1995) Tight regulation, modulation, and high-level expression by vectors containing the arabinose PBAD promoter *J Bacteriol* **177**, 4121-4130
